# Enhanced white matter fiber tract of the cortical visual system in visual artists: implications for creativity

**DOI:** 10.3389/fnins.2023.1248266

**Published:** 2023-10-25

**Authors:** Tzu-Yi Hong, Ching-Ju Yang, Li-Kai Cheng, Wei-Chi Li, Wen-Yih Isaac Tseng, Tzu-Chen Yeh, Hsin-Yen Yu, Li-Fen Chen, Jen-Chuen Hsieh

**Affiliations:** ^1^Institute of Brain Science, College of Medicine, National Yang Ming Chiao Tung University, Taipei, Taiwan; ^2^Integrated Brain Research Unit, Division of Clinical Research, Department of Medical Research, Taipei Veterans General Hospital, Taipei, Taiwan; ^3^Center for Intelligent Drug Systems and Smart Bio-devices (IDS2B), National Yang Ming Chiao Tung University, Hsinchu, Taiwan; ^4^Department of Biological Science and Technology, College of Biological Science and Technology, National Yang Ming Chiao Tung University, Hsinchu, Taiwan; ^5^Institute of Medical Device and Imaging, College of Medicine, National Taiwan University, Taipei, Taiwan; ^6^Department of Radiology, Taipei Veterans General Hospital, Taipei, Taiwan; ^7^Graduate Institute of Arts and Humanities Education, Taipei National University of the Arts, Taipei, Taiwan; ^8^Institute of Biomedical Informatics, College of Medicine, National Yang Ming Chiao Tung University, Taipei, Taiwan; ^9^Brain Research Center, National Yang Ming Chiao Tung University, Taipei, Taiwan

**Keywords:** visual artist, creativity, cortical visual system, diffusion spectrum imaging, tractography, quantitative anisotropy

## Abstract

**Introduction:**

This study aimed to examine the white matter characteristics of visual artists (VAs) in terms of visual creativity and the structural connectivity within the cortical visual system.

**Methods:**

Diffusion spectrum imaging was utilized to examine the changes in white matter within the cortical visual system of a group of VAs (*n* = 25) in comparison to a group of healthy controls matched for age and education (*n* = 24). To assess the integrity of white matter and its relationship with visual creativity, we conducted a comprehensive analysis using region-based and track-specific tractographic examinations.

**Results:**

Our study uncovered that VAs demonstrated increased normalized quantitative anisotropy in specific brain regions, including the right inferior temporal gyrus and right lateral occipital gyrus, along with the corresponding white matter fiber tracts connecting these regions. These enhancements within the cortical visual system were also found to be correlated with measures of visual creativity obtained through psychological assessments.

**Discussion:**

The noted enhancement in the white matter within the cortical visual system of VAs, along with its association with visual creativity, is consistent with earlier research demonstrating heightened functional connectivity in the same system among VAs. Our study’s findings suggest a link between the visual creativity of VAs and structural alterations within the brain’s visual system.

## Introduction

Visual arts encompass a diverse array of creative endeavors, spanning disciplines such as painting, sculpture, ceramics, design, crafts, photography, film, and architecture (Roodhouse, 2006). Visual artists (VAs) cultivate their artistic skills and capabilities through training in various esthetic elements like construction, composition, and abstraction, all with the purpose of bringing their artistic vision and conceptual ideas to life (Lin et al., 2013).

The human cortical visual system consists of two major pathways through which visual information undergoes processing. The dorsal pathway, leading to the parietal lobe, is involved in analyzing spatial relationships and motion, as well as facilitating object-directed actions and visuomotor control (Goodale and Milner, 1992; Kravitz et al., 2013; Freud et al., 2016). This pathway, also known as the “where” route for vision action, provides spatial awareness and information about movement direction (Kravitz et al., 2011). On the other hand, the ventral pathway, also known as the “what” route for vision perception, focuses on identifying objects and tracking their features such as size, shape, and color (Zachariou et al., 2014). This pathway extends from the posterior pole of the occipital cortex to the temporal lobe.

Creativity is a fundamental mental skill in the realm of visual arts (Getzels and Csikszentmihalyi, 2020). The creative aspect of producing visual artwork has been found to activate the inferior temporal gyrus (ITG) in the temporal lobe, which is part of the ventral visual pathway (Kozhevnikov et al., 2013). The ITG plays a crucial role in processing complex visual information related to faces, places, objects, and scenes (Flaherty, 2005; Sugase-Miyamoto et al., 2011; Schaer et al., 2012; Conway, 2018; Beccone, 2020). Through extensive training in the visual arts, this pathway can be strengthened, leading to enhanced coherence in the neural networks associated with creativity (Miller et al., 1996; Petsche, 1996; Jung et al., 2010).

In our previous functional connectivity (FC) study, we found a correlation between the visual creativity of VAs and the strength of intrinsic FC within the two main visual pathways of the cortical visual system (Hong et al., 2023). This observed trait change, known as learning-induced neuroplasticity, can be attributed to the consolidation of neural circuits that are actively engaged during long-term training in the visual arts and esthetic experiences (Hong et al., 2023). More specifically, VAs exhibit enhanced FC with the ITG, which is connected to the visual areas such as the occipital gyrus and cuneus, and this enhanced connectivity appears to be associated with visual creativity (Hong et al., 2023).

Numerous prior studies have demonstrated the efficacy of white matter imaging in tracking changes in the organization of the brain’s white matter, spanning a timeframe of six weeks to nine months (Scholz et al., 2009; Schlegel et al., 2012, 2015). In the current study, we employed the Q-Space diffeomorphic reconstruction (QSDR) approach of diffusion spectrum imaging (DSI) (Yeh et al., 2010; Yeh and Tseng, 2011; Zhang et al., 2013) to investigate the plasticity of fiber tracts between specific regions of the cortical visual system, building upon the work by Hong et al. (2023). The objective of our study was to investigate the structural underpinnings of visual creativity in VAs by analyzing the generalized fractional anisotropy (GFA) and normalized quantitative anisotropy (NQA) of white matter properties. Our findings provide valuable insights into the role of brain structural characteristics in the expression of visual creativity.

## Materials and methods

### Participants

A group of 25 students majoring in VA with an average age of 24.8 ± 1.6, including 5 males, was recruited from Taipei National University of the Arts. These students had received an average of 11.1 ± 4.6 years of specialized visual artistic training. For comparison, 24 healthy controls (CON) of similar age were selected from a local university of arts. The CON group, with a mean age of 23.0 ± 1.8 and 4 males, had no more than 3 years of systematic art training. All participants, in both the VA and CON groups, self-reported as right-handed and had no history of metal implants, brain damage, or neuropsychiatric disorders. Individuals displaying significant emotional instability, as determined by the Beck Depression Inventory (Beck et al., 1996) and Beck Anxiety Inventory (Beck and Steer, 1990) were excluded. Additionally, the Wechsler Abbreviated Scale of Intelligence (WASI-III) (Chen and Chen, 2002) was used to ensure that both groups had comparable levels of general intelligence. This study adhered to the principles of the Declaration of Helsinki and obtained written informed consent from all participants, with approval from the Institutional Review Board of Taipei Veterans General Hospital.

### Psychological measurements

As a subproject within our broader program on neurasthenics (visual art, instrumental arts, dance, singing, percussion, etc.), this study focused on investigating a group of VA and a CON group consisting of non-artist healthy individuals. All artist groups had undergone common psychological measurements and neuroimaging scanning protocols. In this study, the creativity of VA is regarded as a fundamental mental skill, as the process of creating artwork reflects their creativity (Getzels and Csikszentmihalyi, 2020). To evaluate participants’ proficiency in tasks involving visual (figural) and verbal manipulation, all individuals completed the self-reported 40-item Traditional Chinese version of the Abbreviated Torrance Test for Adults (ATTA) (Chen, 2006). The ATTA assesses creative thinking abilities in terms of fluency, originality, elaboration, and flexibility (Chen, 2006). Fluency measures the quantity of ideas generated within a specific time frame, while originality assesses the ability to generate unique ideas. Elaboration evaluates the capacity to elaborate on ideas with details, and flexibility measures the ability to generate a variety of different ideas (Althuizen et al., 2010; Shen and Lai, 2014). The creativity index of the ATTA score is computed by summing the four capacity scores. It is subsequently rescaled using contraction techniques and presented as the creativity level of the ATTA score, which ranges from 1 (minimal) to 7 (substantial) (Althuizen et al., 2010). The tests were conducted and evaluated following established protocols (Chen, 2006).

### Image acquisition

MRI data were collected using a 3 T Siemens Tim Trio MRI System (Siemens Medical, Erlangen, Germany) equipped with a 32-channel head coil at National Yang-Ming University in Taiwan. DSI images offer an advantage over the conventional diffusion tensor imaging technique by enabling the identification of crossing fibers within the white matter fiber bundles of the brain (Martinez-Heras et al., 2021), leading to a more realistic representation of brain network connections (Suo et al., 2021). Quantitative anisotropy (QA) measurement is based on a model-free nonparametric approach, which calculates the density distribution of water diffusion (Yeh and Tseng, 2011). Previous research has demonstrated that QA-aided tractography offers higher resolution compared to fractional anisotropy (FA)-aided and GFA-aided tractography (Yeh et al., 2013). The DSI images were obtained using a spin-echo diffusion echo planar imaging sequence (EPI) with the following parameters: a repetition time (TR) of 9,700 ms, an echo time (TE) of 136 ms, 56 axial slices, a field of view (FOV) of 200 × 200 mm^2^, a matrix size of 80 × 80, a voxel size of 2.5 × 2.5 × 2.5 mm^3^, a bandwidth of 2,156 Hz/Px. The diffusion acquisition scheme consisted of 102 diffusion-encoding directions distributed in a half sphere of diffusion-encoding space (q-space) (Kuo et al., 2008). The maximum b-value was set to 4,000 s/mm^2^, with one image acquired at b = 0 s/mm^2^. To provide anatomical reference for normalization, high-resolution T1-weighted images were obtained using a three-dimensional magnetization prepared rapid gradient echo (3D MPRAGE) sequence. The parameters for the T1-weighted images were as follows: a TR of 2,530 ms, a TE of 3.03 ms, 192 axial slices, a flip angle of 7 degrees, a FOV of 224 × 256 mm^2^, a matrix size of 224 × 256, and a slice thickness of 1 mm. Cushions were used to minimize head movements during the image acquisition process. All images were prescribed in a trans-axial view parallel to the anterior commissure-posterior commissure line. Twelve participants were dropped out and 12 participants were discarded, resulting in a final sample for further analysis.

### Image processing

The DSI data of each participant underwent processing using DSI-Studio.^1^ By employing sampling coordinates, whole-brain QA maps were generated for every voxel. To estimate the QA value for each participant, the QSDR approach and deterministic fiber tractography were applied (Yeh et al., 2010; Yeh and Tseng, 2011; Zhang et al., 2013). QA serves as a fiber-specific metric, quantifying different fiber populations. Additionally, voxel-specific GFA measures, common to all fiber populations within a voxel, were examined. Deterministic fiber tracking was used to map connections between various brain regions. QSDR, a model-free technique based on generalized Q-sampling imaging, calculates the density distribution of water diffusion at different orientations. This is achieved using a high-resolution standard brain atlas constructed from 90-diffusion spectrum imaging datasets in the ICBM-152 space. During the QSDR process, DSI Studio initially computes the QA mapping in the participant’s native space and subsequently normalizes it to the MNI QA map. Furthermore, QSDR records the R-squared value, which represents the goodness-of-fit between the participant’s QA map and the MNI QA map. To ensure comparability across participants and minimize inter-subject variations, the QA values were further normalized by scaling the maximum QA value of each participant to one (termed as normalized QA, NQA). NQA reduces inter-subject spin-density differences and assumes that all the subjects have the same compactness of white matter (Yeh et al., 2019b).

### Region-based and track-specific fiber tractographic analyses and between-group comparisons

To conduct whole-brain tractography, we utilized the DSI-Studio software (see text footnote 1). In this study, a deterministic fiber tracking algorithm (Yeh et al., 2013) was employed, with a QA threshold set at 0.12. The angular threshold was randomly chosen from a range of 15 degrees to 90 degrees. Similarly, the step size was randomly selected between 0.5 voxels and 1.5 voxels. For the seeding process, 1,000,000 points were initiated within the white matter. Tracks that were shorter than 30 mm or longer than 200 mm were discarded. Additionally, an angular threshold of 60 degrees and a step size of 1 mm were applied. To remove false connections, topology-informed pruning, as described by Yeh et al., was implemented (Yeh et al., 2019a).

Next, we conducted region-based and track-specific analyses on each participant, as described by Dhakal et al. (2021). GFA and NQA values were estimated for all possible tracts that traverse six specific brain regions: the right ITG, left ITG, right lateral occipital gyrus (LOG), left LOG, right cuneus, and left cuneus, based on our prior FC study of VA (Hong et al., 2023). To define the regions of interest, we utilized the FreeSurferDKT atlas (Klein and Tourville, 2012). This approach is referred to as region-based tractography. Additionally, we examined the fiber pathways connecting these regions, which is termed as track-specific tractography. From the individual region-based and track-specific fiber tractographic analyses, we extracted the GFA and NQA values. These values were then subjected to group comparisons to identify and characterize the differences in white matter properties between VAs and CONs.

### Correlation analysis

In light of the recognition of VAs as proficient creative practitioners (Degarrod, 2016), we undertook an investigation to examine the correlation between GFA and NQA values derived from region-based and track-specific fiber tractography with the scores on the ATTA scale within both the VA and CON groups. Our primary emphasis was on assessing creativity level, as this metric holistically best represents creative performance and closely aligns with an individual’s standing within the normative data for Taiwan (Chen, 2006).

### Statistical analysis

The statistical analyses for the psychological measurements, region-based and track-specific tractographic metrics, and correlation analyses between the VA and CON groups were conducted using SPSS Statistics (version 23.0, IBM Corp., Armonk, NY). Since artists often exhibit subtler brain structural changes compared to clinical neuropsychiatric patients with prominent brain pathology, we initially employed a more permissive statistical criterion (*p* = 0.05, without correction for multiple tests on seeds and tracts analyzed) to uncover potentially important but understated information. To bolster the reliability of more robust findings, we employed Bonferroni corrections by adjusting the *p*-value. This adjustment involved dividing the *p*-value by the count of seeds and tracts, respectively, examined in region-based and track-specific tractographic analyses. Nonetheless, employing the Bonferroni correction method may lead to the inadvertent neglect of subtle effects. In exploratory and preliminary research, the primary objective typically revolves around generating hypotheses and uncovering potential trends or patterns that can serve as foundations for future research endeavors (Fife, 2020). To achieve a harmonious approach in our exploratory and preliminary research, we have opted to present both unadjusted *p*-values and correction adjusted *p*-values in our findings.

## Results

### Demographic data and psychological assessments

No significant differences were observed between the groups in terms of age, gender, and intelligence (Table 1). However, when compared to the CON group, the VA group exhibited significantly higher scores in visual (figural) creativity (VA: 5.32 ± 2.75, CON: 3.03 ± 2.26, *p* < 0.001), fluency (VA: 16.44 ± 1.58, CON: 14.96 ± 1.55, *p* = 0.045), elaboration (VA: 17.68 ± 1.79, CON: 15.9 ± 2.8, *p* = 0.004), and flexibility (VA: 15.36 ± 1.7, CON: 14.5 ± 1.6, *p* = 0.048). The VA group also had higher ATTA creativity index (VA: 73.0 ± 6.13, CON: 66.29 ± 8.35, *p* < 0.001) and ATTA creativity level (VA: 5.56.0 ± 1.25, CON: 4.25 ± 1.46, *p* < 0.001) compared to the CON group. It is worth noting that the VA group showed a trend toward higher originality performance compared to the CON group, although this difference was not statistically significant (VA: 17.24 ± 2.2, CON: 15.8 ± 2.6, *p* = 0.077) (Table 1).

**Table 1:** Demographic data and psychological results.

	VAs	CONs	*p* value
	(*n* = 25)	(*n* = 24)	
Age (years)	24.8 ± 1.6	23.0 ± 1.8	0.38
Sex (male/female)	5/20	4 /20	0.67
Duration of learning (years)	11.1 ± 4.6	–	–
Education (years)	16.8 ± 1.6	16.3 ± 1.2	0.14
WAIS-III	110.97 ± 7.38	109.51 ± 7.22	0.55
ATTA Verbal creativity	1.10 ± 0.9	0.67 ± 0.76	0.54
Visual creativity	5.32 ± 2.75	3.03 ± 2.26	< 0.001***
Fluency	16.44 ± 1.58	14.96 ± 1.55	0.045*
Originality	17.24 ± 2.2	15.8 ± 2.6	0.077
Elaboration	17.68 **±** 1.79	15.9 **±** 2.8	0.004**
Flexibility	15.36 ± 1.7	14.5 ± 1.6	0.048*
Creativity index	73.0 ± 6.13	66.29 ± 8.35	< 0.001***
Creativity level	5.56 ± 1.25	4.25 ± 1.46	< 0.001***

Data expressed as mean ± standard deviation. *p < 0.05, **p < 0.01, ***p < 0.001; VA, visual artist; CON, control; WAIS-III, Wechsler Adult Intelligence Scale-III; ATTA: Abbreviated Torrance Test for Adults.

### Region-based fiber tractography comparisons between VA and CON groups

**Figure 1:**
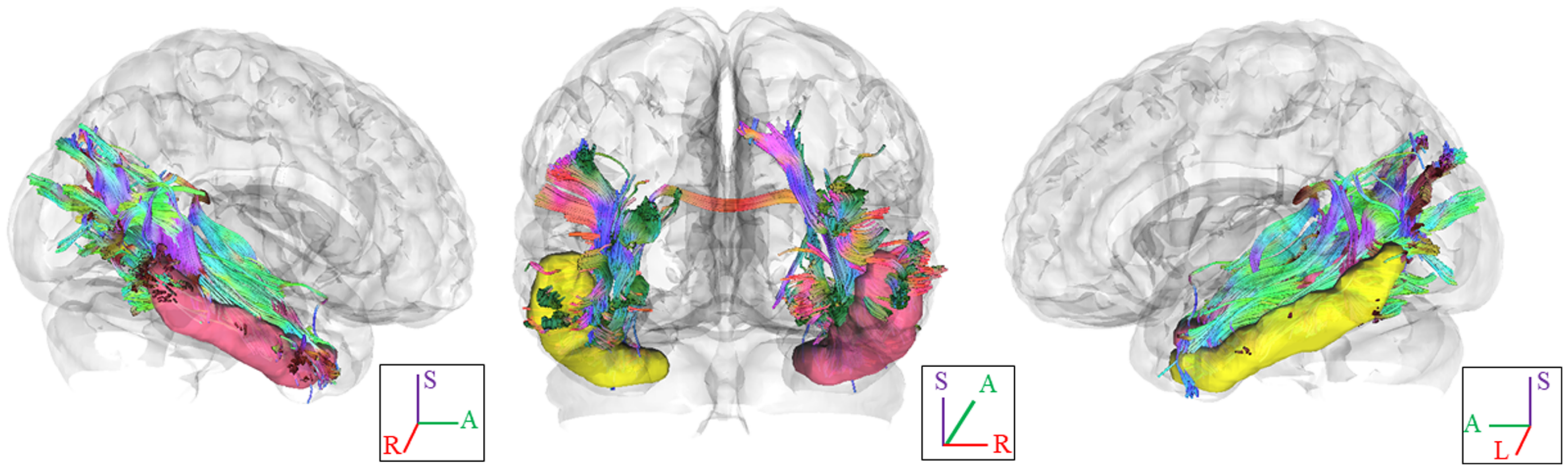
Fiber pathways through the ITG seed region in each hemisphere for a representative participant. In this visual depiction, fibers are color-coded to represent their orientation: *red* indicates fibers along the *X*-axis (left–right), *green* indicates fibers along the *Y*-axis (anterior–posterior), and *blue* indicates fibers along the *Z*-axis (inferior–superior). R, right; L., left; (S), superior; (A), anterior; ITG, inferior temporal gyrus.

**Figure 2:**
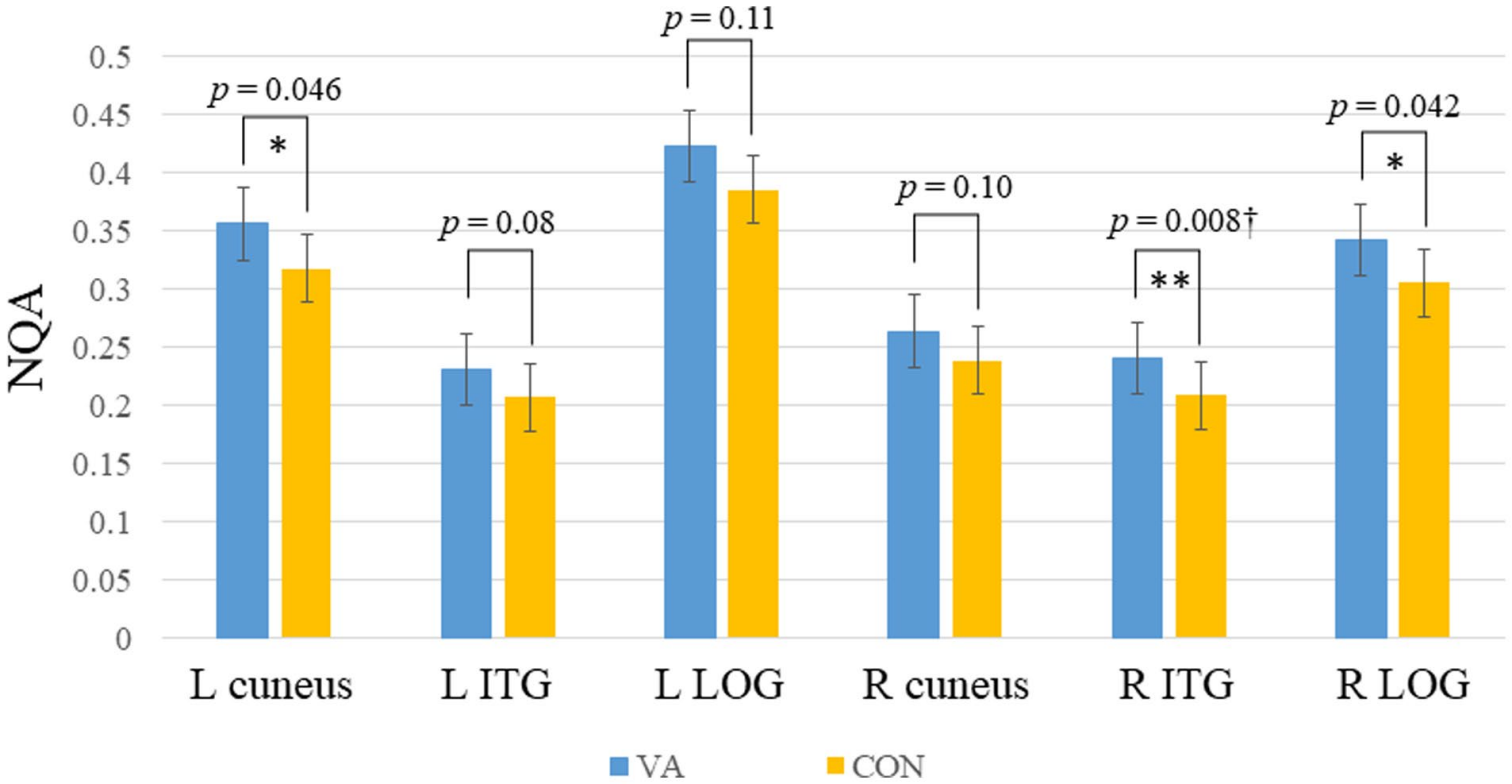
Between-group region-based tractography difference. The VAs group exhibited significantly higher NQA measures in the L cuneus, R ITG, and R LOG. *p <0.05; **p<0.01 (unadjusted). †denotes significant after Bonferroni’s correction (*p* = 0.0083, number of seeds analyzed is 6). Error bars represent the standard error of the mean. NQA, normalized quantitative anisotropy; VA, visual artist; CON, control; L, left; R, right; ITG, inferior temporal gyrus; LOG, lateral occipital gyrus.

In Figure 1, we provide an illustration of fiber tracts passing through the right and left ITG region for a representative participant. Significant differences were observed in region-based tractographic NQA values within the left cuneus, right ITG, and right LOG when comparing the VA and CON groups (Figure 2). However, there were no significant differences in GFA values between the two groups. Importantly, the VA group exhibited higher NQA values in regions associated with visually guided spatial memory, visual–spatial processing, and navigation.

### Track-specific fiber tractography comparisons between VA and CON groups

**Figure 3:**
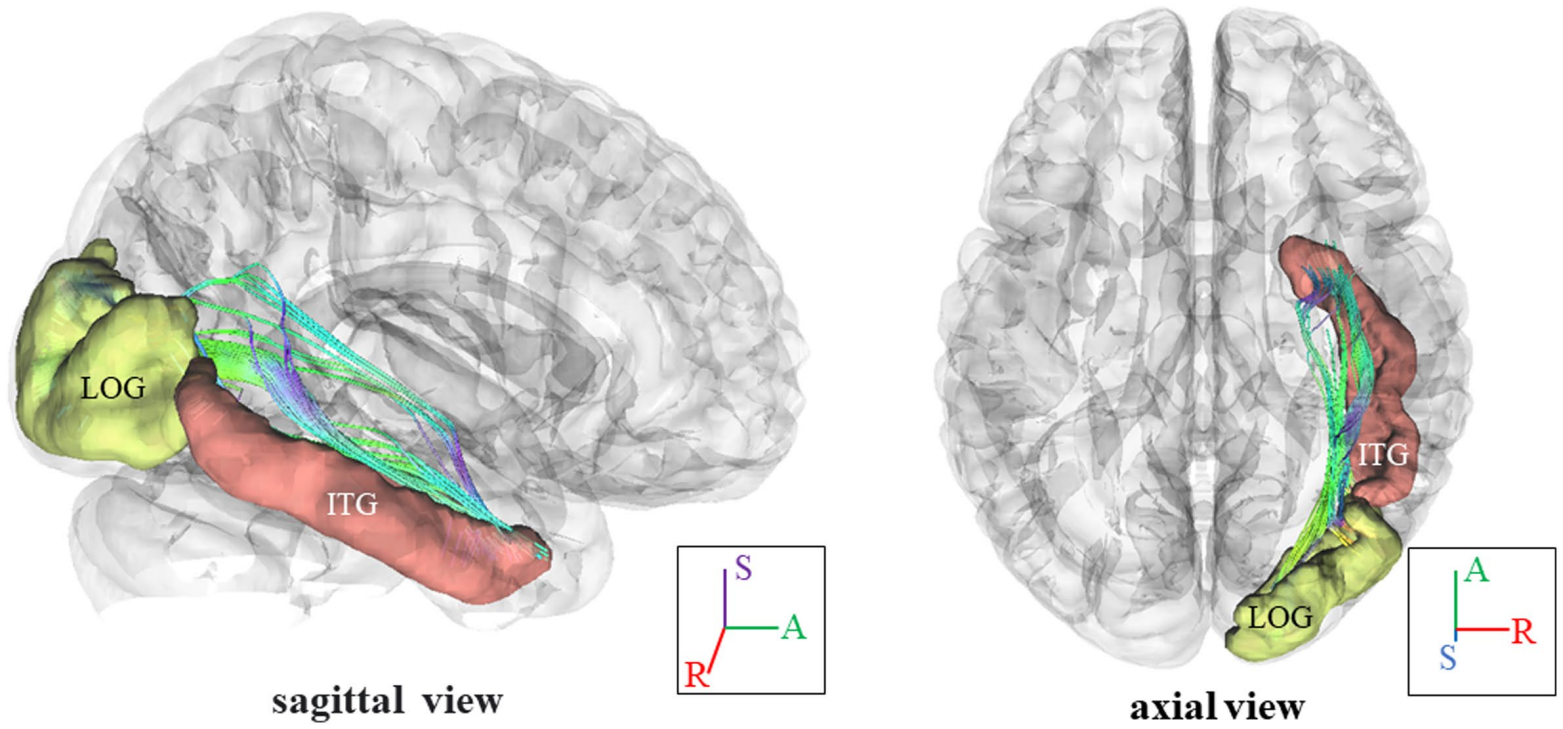
Fiber pathways in the right ILF between right ITG and right LOG for a representative participant. In this visual depiction, fibers are color-coded to represent their orientation: *red* indicates fibers along the *X*-axis (left–right), *green* indicates fibers along the *Y*-axis (anterior–posterior), and *blue* indicates fibers along the *Z*-axis (inferior–superior). R, right; (S), superior; (A), anterior; ILF, inferior longitudinal fasciculus; ITG, inferior temporal gyrus, LOG, lateral occipital gyrus.

**Figure 4:**
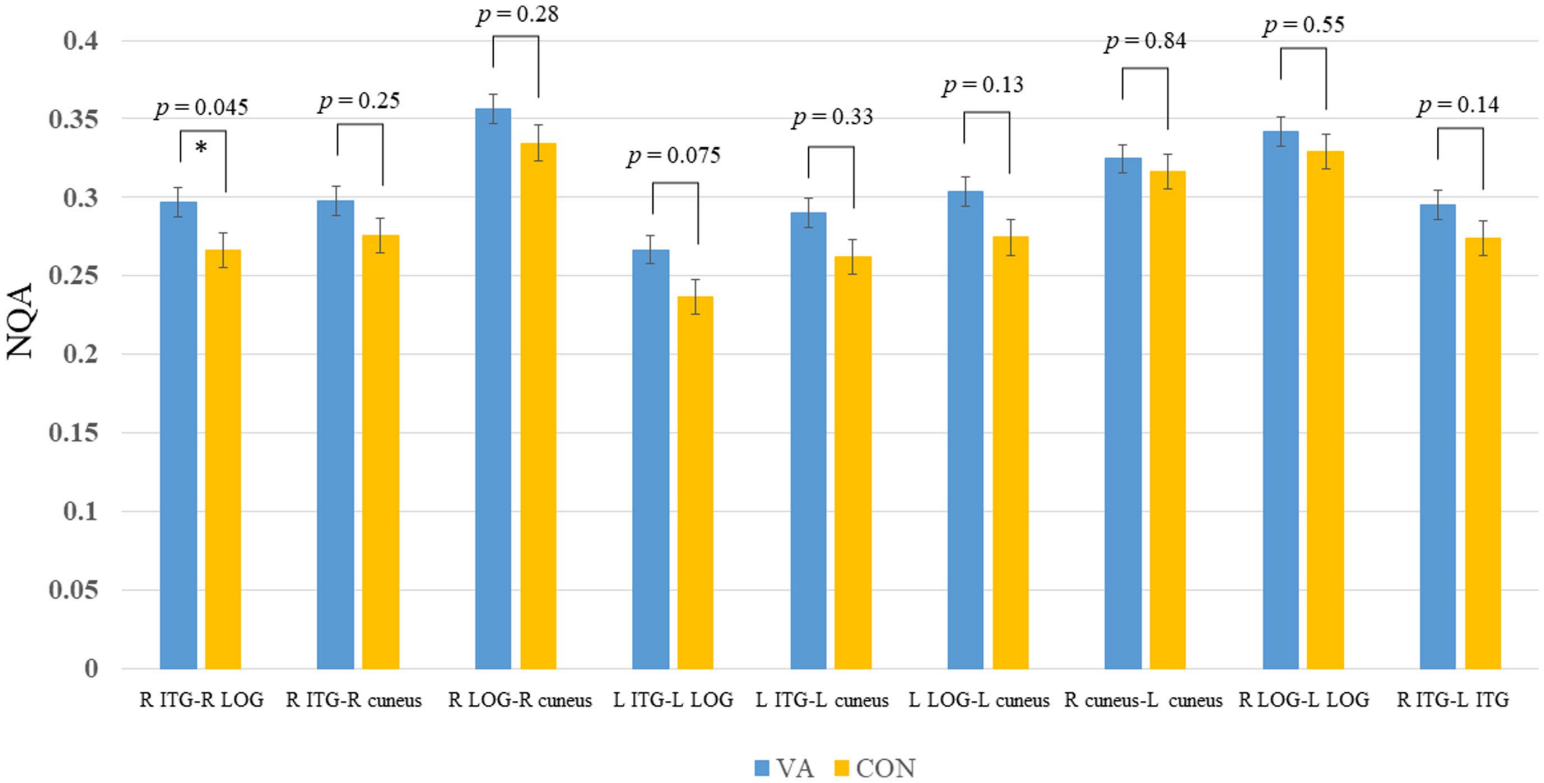
Between-group track-specific tractography difference. The VA group exhibits significantly higher NQA measures in the R ILF between the R ITG and R LOG. Error bars represent the standard error of the mean. *p <0.05 (unadjusted). The permissive significance level does not meet the rigorous adjustment (*p* = 0.0056, number of tracts analyzed is 9). NQA, normalized quantitative anisotropy; VA, visual artist; CON, control; R, right; L, left; ILF, inferior longitudinal fasciculus; ITG, inferior temporal gyrus; LOG, lateral occipital gyrus.

In Figure 3, we present the fiber pathway(s) that connect the right ITG and right LOG for a representative participant. Our results, depicted in Figure 4, reveal a significant difference in NQA within the pathway linking the right ITG and LOG, which corresponds to the right inferior longitudinal fasciculus (ILF) according to the Human Connectome Project diffusion MRI template (HCP842 tractography atlas) (Yeh et al., 2018), between the VA and CON groups. However, no significant differences in GFA were observed between the two groups.

### Differential correlation between NQA in the right ILF and ATTA scores

**Figure 5:**
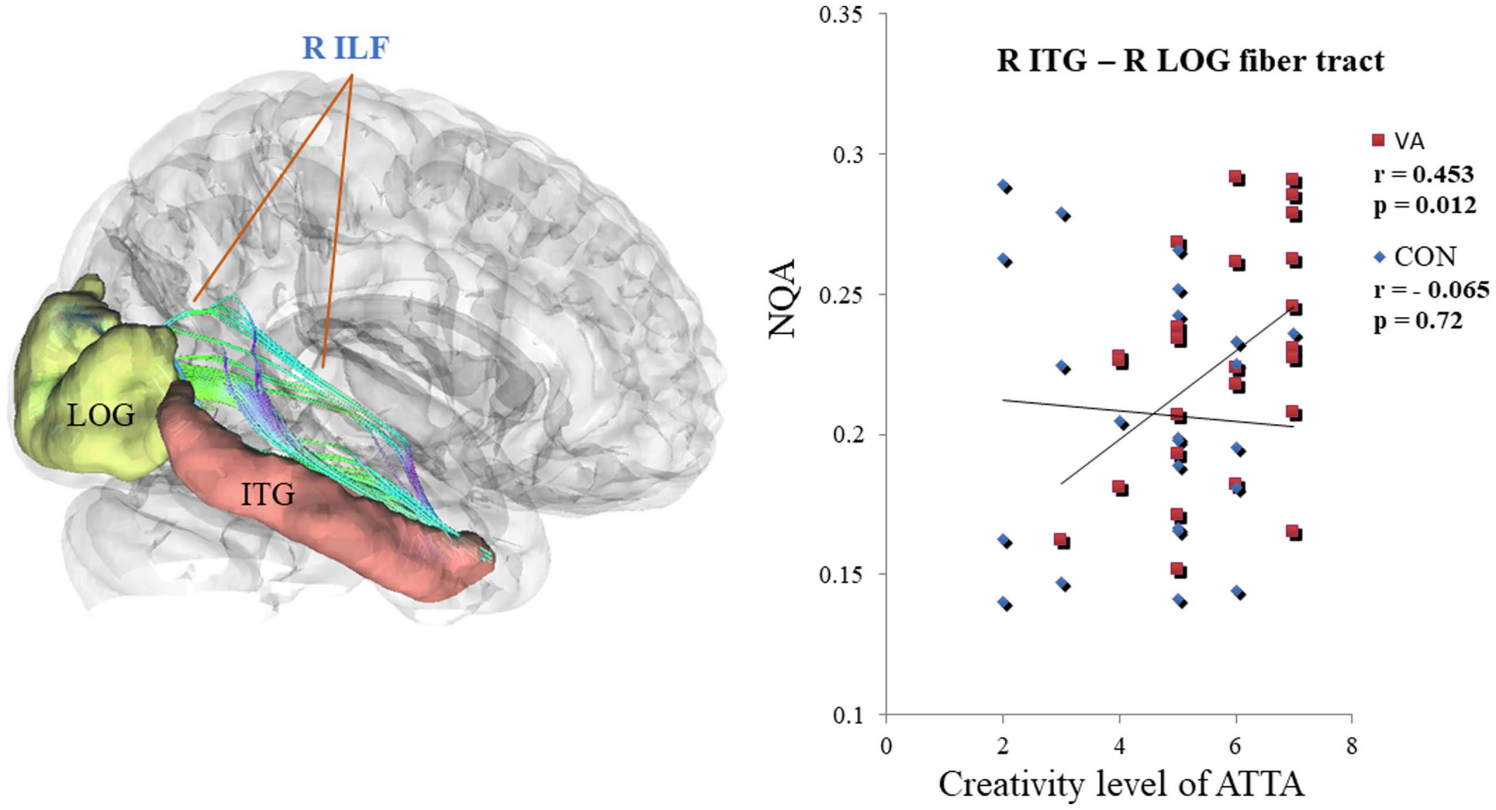
Differential Correlation Between NQA in the R ILF and the creativity level of ATTA Scores in VAs and CONs. In the R ILF, the strength of NQA between the R ITG and the R LOG exhibits a positive correlation with the creativity level of ATTA scores in VAs. Conversely, the strength of NQA in the R ILF does not show any correlation with creativity level of ATTA scores in CONs. NQA, normalized quantitative anisotropy; R, right; ILF, inferior longitudinal fasciculus; ATTA, Abbreviated Torrance Test for Adults; VA, visual artist; CON, control; ITG, inferior temporal gyrus; LOG, lateral occipital gyrus.

Within the VA group, a noteworthy positive correlation (VA, *r* = 0.453, *p* < 0.05) emerged between creativity level and NQA in the right ILF, connecting the right ITG and right LOG regions. In contrast, the CON group exhibited no significant correlation (CON, *r* = −0.065, *p* = 0.72) (as depicted in Figure 5). Furthermore, upon closer scrutiny, no significant correlations were observed between white matter metrics (specifically, GFA and NQA values derived from region-based and track-specific fiber tractography) and various other ATTA subscores, including fluency, originality, elaboration, and flexibility, within both the VA and CON groups.

## Discussion

In our investigation of the effects of long-term visual artistic training on white matter plasticity, we employed region-based and track-specific fiber tractography methods. The results revealed an enhancement in fiber integrity among VAs in specific fiber tracts that traverse the right ITG, right LOG, and left cuneus (Figure 2). Notably, the most pronounced enhancement was observed in the ILF, which connects the right ITG and right LOG, as depicted in Figure 4. It is worth mentioning that the degree of enhancement in the ILF showed a positive correlation with the creativity level measured by the ATTA scale in VAs, as shown in Figure 5. These structural improvements have the potential to enhance essential visuospatial abilities utilized by visual artists during the creative process, as suggested by (Winner et al., 1991).

### Enhancing white matter integrity through long-term visual artistic training

Neuroplasticity in the human brain refers to the remarkable ability of neural networks to restructure and adapt in response to various factors, including experience, injury, learning, and healing (Sampaio-Baptista and Johansen-Berg, 2017). Specifically, white matter plasticity, which is influenced by neural activity and learning (Stevens et al., 1998; Ishibashi et al., 2006; Gibson et al., 2014; McKenzie et al., 2014; Fields, 2015), involves the restructuring of neural networks by enhancing myelination, axon diameter, internode length, and ion channel density (Zatorre et al., 2012). These changes contribute to the improved efficiency of transmitting action potentials (Takeuchi et al., 2010; Sampaio-Baptista and Johansen-Berg, 2017; Frizzell et al., 2020; Wu et al., 2021; Kirby et al., 2022).

NQA, which reflects the structural properties of white matter, is influenced by various factors including axon density, axon size, and myelination of axons and glial cells (Zatorre et al., 2012). Higher levels of NQA indicate that long-term visual artistic training may be associated with increased axon density and myelination in white matter. In the context of long-term visual artistic training, our investigations have revealed that visual VAs exhibit enhanced fiber tracts in their brains. These augmented fiber tracts potentially form the foundation for the proficient execution of the skills and strategies acquired through artistic training and learning, enabling the creative production of artwork (Brown and Kim, 2021). This process necessitates optimal cognitive engagement. The structural architecture of visual artists thus plays a critical role in facilitating their functional interactions for the purpose of creative artistic expression.

### Creative skills linked to adaptive white matter alterations in VAs

The VA group demonstrated higher levels of creativity, as indicated by their elevated ATTA scores, compared to the CON group (Table 1). In the VA group, we observed a significant positive correlation between the creativity level, as measured by the ATTA score, and the NQA values of the ILF connecting the right ITG with the right LOG (Figure 5). The production of artworks heavily relies on the cortical visual system, particularly the ITG located in the ventral pathway. The ITG plays a crucial role in object, face, and scene perception (Conway, 2018). It is also involved in visual creativity, encompassing visual imagery, visual perception (Ishai et al., 2000), and visual attention for object recognition (Zhang et al., 2011). Our findings suggest that the ventral pathway is strengthened and consolidated in individuals with visual artistic training, as evidenced by the increased NQA between the ITG and LOG resulting from long-term practice in translating artistic concepts into visual form. The current fiber tractographic study reinforces the findings of our previous research on the FC of the cortical visual system in VAs (Hong et al., 2023). In our prior study, we demonstrated a positive correlation between visual creativity and increased FC between the ITG and occipital cortices of the ventral pathway (Hong et al., 2023). The present findings provide further support for these observations. Taken together, these results suggest that long-term training in visual arts may contribute to the strengthening of the ventral pathway and the establishment of white matter structural connectivity associated with visual creativity. The strength of white matter structural connectivity has the potential to be linked to visual creativity.

### Challenges in using GFA to detect white matter alterations in skilled artists

Our investigation utilizing region-based and track-specific fiber tractography analysis yielded no substantial GFA variations. This outcome can be attributed to the limitations of GFA, which is susceptible to the partial volume effect and exhibits value reduction in the presence of fiber crossing or voxels affected by the partial volume effect (Yeh et al., 2010, 2013). To support our findings, we refer to a previous study that employed a similar DSI approach (using NQA and GFA to study white matter properties) to explore white matter plasticity in jazz improvisers (Dhakal et al., 2021). This further reinforces the lack of efficacy in utilizing GFA for detecting white matter alterations in skilled artists.

### Limitations and future direction

The current study possesses certain limitations. Firstly, our evaluation of creativity was solely based on the ATTA measure, which predominantly captures general creativity. To obtain a more comprehensive understanding, future investigations should incorporate additional task variations that specifically target different aspects of visual creativity. This approach will facilitate a more in-depth exploration of the various psychological factors involved in long-term training in the visual arts. Secondly, our study exclusively focused on the cortical visual system, considering its fundamental role in visual arts. However, it would be valuable for future researchers to conduct a more extensive brain-wise analysis of network properties and structural architecture (Bullmore and Sporns, 2009; Yeh, 2020) in order to better characterize the white matter networks associated with the identified neural substrates of visual creativity (De Pisapia et al., 2016; Pidgeon et al., 2016; Zhu et al., 2017). This expanded analysis would contribute to a better understanding of the central mechanisms underlying visual creativity. Finally, it’s imperative to view this report as an exploratory and preliminary study, given our approach of using both permissive and stringent statistical methods to present and discuss the findings with the goal of uncovering potentially valuable insights that can guide future investigations. It is noteworthy that structural brain changes in artists may not be as conspicuous as those observed in clinical patients with pronounced neuropsychiatric and bio-behavioral symptoms, where such brain alterations are more apparent. Therefore, we advocate for future research to uphold robust statistical standards by incorporating larger sample sizes.

## Conclusion

In conclusion, our study indicates that long-term visual artistic training has an impact on axon myelination, leading to enhanced efficiency in the creation of artwork. This study reveals a structural neuroplasticity in the cortical visual system that is induced by training and corresponds to the skilled performance observed in visual artists.

## Data availability statement

The raw data supporting the conclusions of this article will be made available by the authors, without undue reservation.

## Ethics statement

The studies involving humans were approved by the Institutional Review Board of Taipei Veterans General Hospital. The participants provided their written informed consent to participate in this study.

## Author contributions

L-FC and J-CH incepted and designed the experiment. T-YH, L-KC, C-JY, and W-CL performed the experiments. T-YH analyzed the data. H-YY, W-YT, and T-YH contributed materials. L-FC and T-CY for discussion inputs. T-YH and J-CH wrote the paper. All authors had reviewed the paper. J-CH approved the final submission. All authors contributed to the article and approved the submitted version.

## Footnotes

^1^http://dsi-studio.labsolver.org/
